# 3D automatic liver and spleen assessment in predicting overt hepatic encephalopathy before TIPS: a multi-center study

**DOI:** 10.1007/s12072-023-10570-5

**Published:** 2023-08-02

**Authors:** Xiaoqiong Chen, Tao Wang, Zhonghua Ji, Junyang Luo, Weifu Lv, Haifang Wang, Yujie Zhao, Chongyang Duan, Xiangrong Yu, Qiyang Li, Jiawei Zhang, Jinqiang Chen, Xiaoling Zhang, Mingsheng Huang, Shuoling Zhou, Ligong Lu, Meiyan Huang, Sirui Fu

**Affiliations:** 1 Zhuhai Interventional Medical Centre, Zhuhai Hospital Affiliated with Jinan University (Zhuhai People’s Hospital), No. 79 Kangning Road, Zhuhai, 519000 Guangdong Province China; 2 Zhuhai Engineering Technology Research Center of Intelligent Medical Imaging, Zhuhai Hospital Affiliated with Jinan University (Zhuhai People’s Hospital), Zhuhai, China; 3 School of Biomedical Engineering, Southern Medical University, No. 1023-1063 Shatai Road, Guangzhou, 510515 Guangdong China; 4 Department of Anesthesia, Shanghai East Hospital, Tongji University School of Medicine, Shanghai, China; 5 Department of Interventional Radiology, The Third Affiliated Hospital of Sun Yat-sen University, Guangzhou, China; 6 Interventional Radiology Department, The First Affiliated Hospital of USTC, Division of Life Sciences and Medicine, University of Science and Technology of China, Hefei, China; 7 Department of Laboratory Medicine, Nanfang Hospital, Southern Medical University, Guangzhou, China; 8 Department of Biostatistics, School of Public Health, Southern Medical University, Guangzhou, China; 9 Department of Radiology, Zhuhai Hospital Affiliated with Jinan University (Zhuhai People’s Hospital), Zhuhai, China; 10 Department of Radiology, Shenzhen People’s Hospital, Shenzhen, China; 11 Guangdong Provincial Key Laboratory of Medical Image Processing, Southern Medical University, Guangzhou, China; 12 Guangdong Province Engineering Laboratory for Medical Imaging and Diagnostic Technology, Southern Medical University, Guangzhou, China

**Keywords:** Transjugular intrahepatic portosystemic shunt, Overt HE, 3D assessment, 2D factors, 3D factors, Prediction, 2D model, 3D model, Optimal model, Applet

## Abstract

**Background:**

Overt hepatic encephalopathy (HE) should be predicted preoperatively to identify suitable candidates for transjugular intrahepatic portosystemic shunt (TIPS) instead of first-line treatment. This study aimed to construct a 3D assessment-based model to predict post-TIPS overt HE.

**Methods:**

In this multi-center cohort study, 487 patients who underwent TIPS were subdivided into a training dataset (390 cases from three hospitals) and an external validation dataset (97 cases from another two hospitals). Candidate factors included clinical, vascular, and 2D and 3D data. Combining the least absolute shrinkage and operator method, support vector machine, and probability calibration by isotonic regression, we constructed four predictive models: clinical, 2D, 3D, and combined models. Their discrimination and calibration were compared to identify the optimal model, with subgroup analysis performed.

**Results:**

The 3D model showed better discrimination than did the 2D model (training: 0.719 vs. 0.691; validation: 0.730 vs. 0.622). The model combining clinical and 3D factors outperformed the clinical and 3D models (training: 0.802 vs. 0.735 vs. 0.719; validation: 0.816 vs. 0.723 vs. 0.730; all *p* < 0.050). Moreover, the combined model had the best calibration. The performance of the best model was not affected by the total bilirubin level, Child–Pugh score, ammonia level, or the indication for TIPS.

**Conclusion:**

3D assessment of the liver and the spleen provided additional information to predict overt HE, improving the chance of TIPS for suitable patients. 3D assessment could also be used in similar studies related to cirrhosis.

**Graphical abstract:**

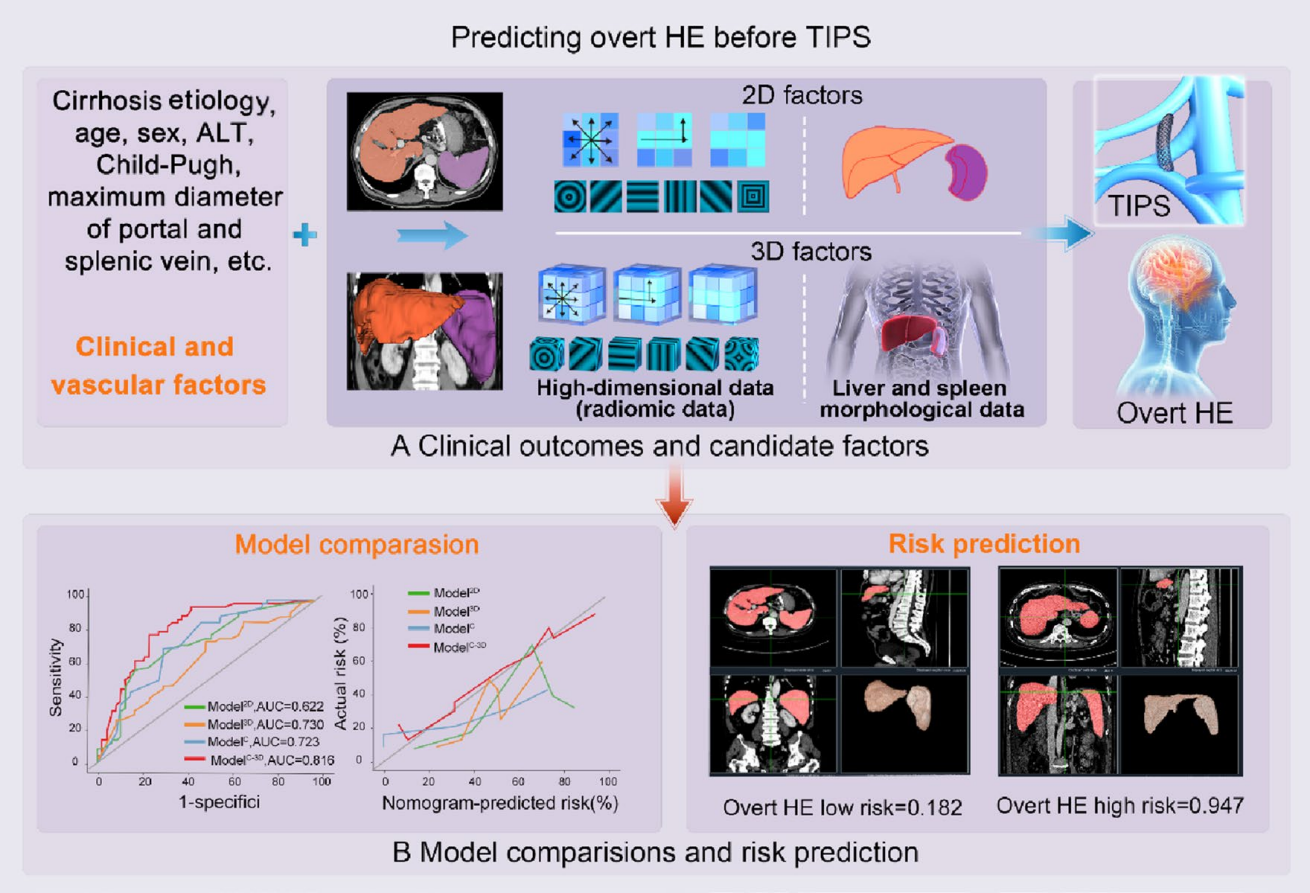

**Supplementary Information:**

The online version contains supplementary material available at 10.1007/s12072-023-10570-5.

## Introduction

Portal hypertension due to cirrhosis can lead to variceal bleeding and refractory ascites [1]. Transjugular intrahepatic portosystemic shunt (TIPS), which establishes a channel between the portal and hepatic veins, is one of the alternative treatments for variceal bleeding and ascites [2, 3]. However, American, Chinese, and European guidelines recommend endoscopic therapy, non-selective β-blockers, and paracentesis for portal hypertension-related variceal bleeding and ascites; TIPS is considered an alternative option [4–7]. This is because TIPS can cause overt hepatic encephalopathy (HE), negatively impacting patients’ quality of life and increasing mortality [2, 8]. Post-TIPS overt HE incidence ranges from 10 to 50% [9]. Moreover, considering that overt HE may reoccur, even after multiple treatments, a more reasonable decision making method is to identify the low-risk population prior to TIPS [10]. By stratifying patients for risk of post-TIPS overt HE, suitable patients for TIPS can be selected.

Several clinical and biological factors are related to overt HE, such as the Child–Pugh score [11] and interleukin-6 levels [12]. Recently, conventional computed tomography (CT) imaging has attracted attention in cirrhosis-related diseases [13, 14]. Some researchers focus on morphological assessments of the liver, such as the liver surface nodularity score [15]. However, to take full advantage of the abovementioned methods, a 3D assessment may outperform 2D ones by avoiding bias from slice selection and increasing assessment comprehensiveness. More recently, deep learning methods based on convolutional neural networks have been widely used in medical image segmentation tasks [16–18], such as U-Net. Among them, nnU-Net, a self-adjusting framework designed from U-Net, has achieved impressive performance in many segmentation tasks, including pancreas and kidney segmentation [19]. Therefore, if 3D segmentation by nnU-Net is possible, the morphological and high-dimensional data can be extracted in 3D to achieve a more precise model to predict post-TIPS overt HE.

Considering the significance of overt HE in patient selection for TIPS, this study involved clinical challenge identification, data collection, model comparison, and model interpretation. We aimed to construct a 3D assessment-based model to predict post-TIPS overt HE.

## Materials and methods

### Patient selection

**Fig. 1:**
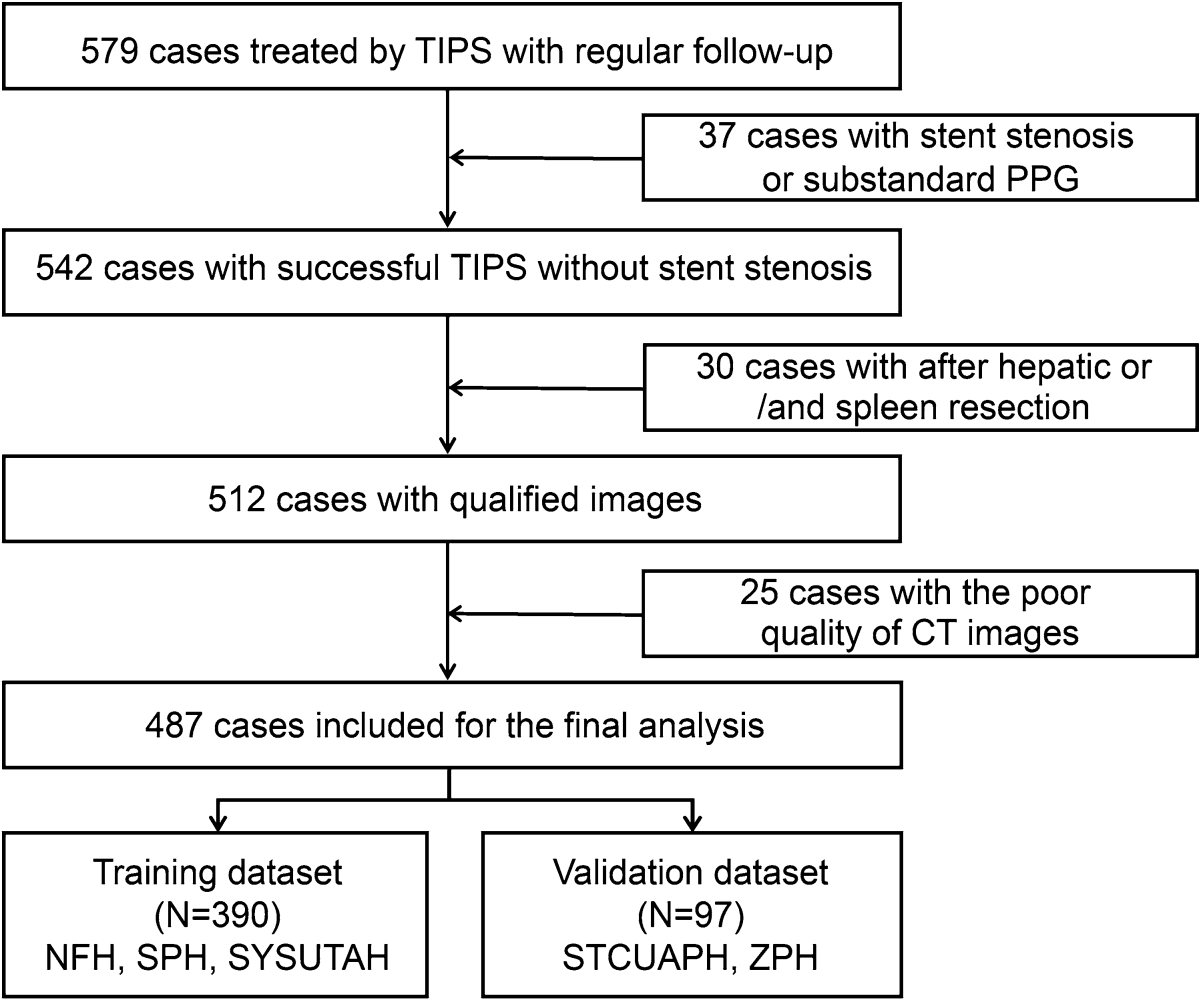
Inclusion and exclusion flowcharts

Patients treated with TIPS between January 2012 and January 2021 were identified. Data was collected from five hospitals in China: Nanfang Hospital (NFH), Shenzhen People’s Hospital (SPH), The Third Affiliated Hospital of Sun Yat-sen University (SYSUTAH), The First Affiliated Hospital of the University of Science and Technology of China (STCUAPH), and Zhuhai People’s Hospital (ZPH). All patients underwent TIPS treatment for variceal rebleeding and/or refractory ascites. The inclusion criteria were: (1) regular follow-up for at least 1 year; (2) at least one variceal rebleeding or refractory ascites after therapies such as endoscopic treatment, vasoactive drugs, or large-volume paracentesis; (3) TIPS performed by puncture from the right hepatic vein to the bifurcation of the left and right branches of the portal vein; (4) Child–Pugh score ≤ 13 points; (5) ≥ 18 years old; (6) a portosystemic pressure gradient (PPG) decrease < 50% from baseline or < 12 mmHg after TIPS [5, 7]. The exclusion criteria were: (1) TIPS performed to prevent failure or rebleeding after initial pharmacological and endoscopic therapy (early TIPS); (2) hepatocellular carcinoma that did not accord with the Milan criteria for transplantation i.e., a single lesion < 3 cm or fewer than three lesions with the largest measuring ≤ 3 cm (per the Milan criteria [20], an HCC patient is eligible for liver transplantation if they have either a single lesion ≤ 5 cm in size or two to three lesions, each ≤ 3 cm in size); (3) stent stenosis or occlusion during follow-up; (4) liver cancer on the liver surface hampering liver depression evaluation; (5) liver and/or spleen resection did not acquire total liver and/or spleen volume. Ultimately, 487 cases were enrolled in our study (Fig. 1).

The study protocols were approved by the Ethics Review Committee of the Zhuhai People’s Hospital. Informed consent was waived because the patients’ data were collected retrospectively. All patient data were anonymized before analysis.

### Preoperative treatment

Based on the guidelines, the following preoperative treatments were performed if necessary: (1) anemia and coagulopathy were corrected to ensure patient safety during TIPS treatment (hemoglobin > 7 g/dL and prothrombin time < 25 s); (2) abdominal paracentesis was performed before TIPS to prevent massive hemorrhage; (3) vasoactive drugs (terlipressin [2 mg/4 h], somatostatin [250 to 500 μg/h] or octreotide [25 to 50 μg/h]), and prophylactic antibiotics (ceftriaxone [1 g/24 h]) were administered before the TIPS [2].

### Procedures of TIPS

All the procedures were performed by physicians with at least 10 years of experience in interventional radiology. The procedure was performed as follows: (1) after general anesthesia, the bifurcation of the main trunk and the left and right branches of the portal vein was punctured from the right hepatic vein, and the preoperative PPG was measured before stent deployment; (2) before stent implantation a 6-mm balloon was used to expand the puncture channel and an 8-mm polytetrafluoroethylene-covered stent was implanted; (3) to prevent stent dilation after TIPS, an 8-mm balloon was used to perform dilatation again to ensure that the stent was expanded to 8 mm; (4) after stent insertion, petrography was performed to enable visualization of the left and right branches of the portal vein; and, (5) finally, postoperative PPG was measured. PPG reductions of more than 50% from baseline or < 12 mmHg were considered successful [2].

### Follow-up and outcomes

Following the guidelines, patients were not given oral medicines, such as lactulose or rifaximin, after TIPS until HE occurred. None of the patients received any pharmacological treatment to prevent the occurrence of HE [9]. For the included patients, the baseline demographic characteristics and CT images were collected within 7 days before the TIPS. Follow-up was performed once per week in the outpatient department for the first month; subsequently, follow-up, including telephone interviews, outpatient visits, or hospital visits, was scheduled every 4 weeks. Patients and their families were asked to contact a physician immediately upon any alteration in the patient’s mental state. HE symptoms such as lethargy, apathy, and obvious personality changes were recorded in detail. After repeated confirmation, the stage and degree of HE were evaluated.

The outcome of this study was post-TIPS overt HE, defined as grades II, III, or IV according to the West Haven Criteria (detailed in Supplementary Table 1) [9]. For patients without overt HE, follow-up was continued every 4 weeks until liver transplantation, death, or the end of the study (July 2022).

### Image acquisition and 3D segmentation

**Fig. 2:**
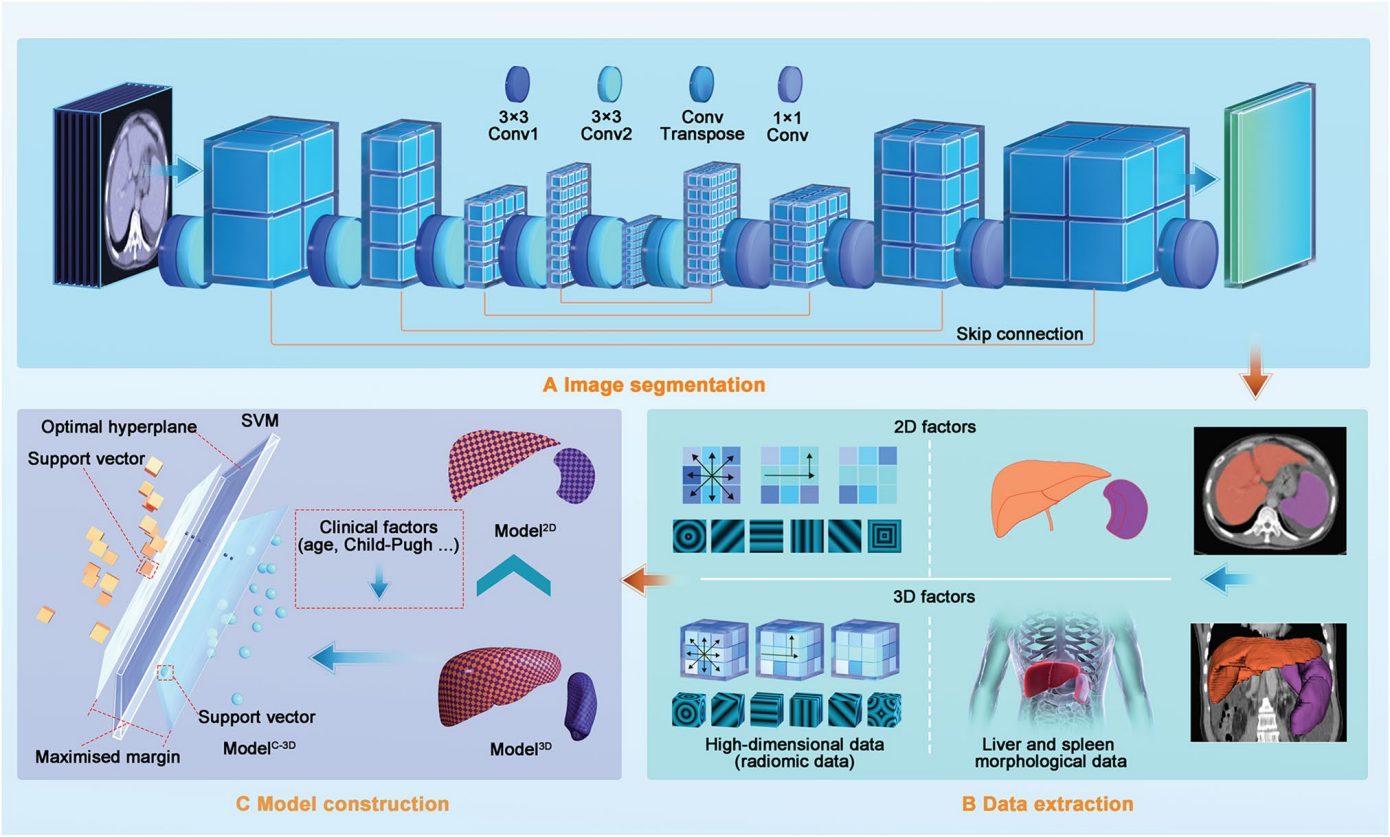
Workflow for model construction. Workflow for model construction. **a** Segmentation of liver and spleen on CT images using nnU-Net. **b** After extracting 2D and 3D factors (including morphologic and high-dimensional factors), we constructed 2D and 3D models. **c** Since the 3D model performed better than did the 2D model, we used the clinical and 3D factors to construct the combined model using the support vector machine. *CT* computed tomography

The CT scanning and contrast agency injection parameters in each collaborative hospital are listed in Supplementary Table 2. As the veins had a clearer boundary in the portal phase than in other phases, we used the CT images of the portal phase for analytical imaging. CT scan coverage ranged from the dome to the lower edges of the liver and the spleen. The image data was transferred directly to the picture archiving and communication system. All the image data were exported in DICOM format for image 3D segmentation and data extraction. The nnU-Net, a popular medical image segmentation framework, improved from U-Net [21], and was used to achieve automatic liver and spleen segmentation. Moreover, a dynamic training strategy was used to ensure segmentation performance (detailed in Supplementary Text 1 and Fig. 2a).

### Data extraction

Candidate clinical factors are listed in Supplementary Table 3. To assess the morphologic changes of the liver and the spleen, we used two categories of parameters. The first was related to changes in diameter or volume, such as the maximum diameter of the liver or the spleen volume (Supplementary Table 4). To control the bias introduced by the physiological differences between individuals, we used the diameters of the portal and splenic vasculature to standardize them. Considering the complexity and variability of the portal and splenic vasculature, we used the previously reported method to assess morphological changes of the vasculature [22, 23], such as the ratio of the maximum diameters of the liver and the portal vein (Supplementary Table 3). Both the original and standardized parameters were included in the analysis to test the necessity of standardization. The second category was related to changes in CT attenuation. Similarly, self-comparison (such as the Mean CT attenuation ratio between the liver and the spleen) was used to control individual-related bias, besides original mean value-related parameters (Supplementary Table 4).

The second category included high-dimensional factors, which may also provide useful information. Since radiomics could capture quantitative imaging features which reflected the underlying tissue characteristics [24–26], we used radiomics to extract high-dimensional information. In total, we extracted 863 3D features and 479 2D features of the liver and the spleen, including shape-based, histogram, and textural features (Fig. 2b).

### Statistical analysis

To confirm the generalization ability of the prediction model, external validation was performed in this study. The patients from three centers, NFH, SPH, and SYSUTAH, were considered training datasets, while the remaining patients from the other two centers, STCUAPH and ZPH, were used as validation datasets. Quantitative data are expressed as means (standard deviations) or medians (interquartile ranges) based on their distribution. Distributions between groups were compared using the t-test or Wilcoxon rank sum test, as appropriate. Similarly, categorical variables are displayed as percentages, compared using Pearson’s Chi-squared test or Fisher’s exact test.

After dividing the patient data into training and external validation datasets, first, we used the least absolute shrinkage and selection operator (LASSO) regression for preliminary screening of the clinical and 2D and 3D data (including morphologic and high-dimensional data). The data that passed the screening were further selected by the grid search method and tenfold cross-validation. Second, we used the support vector machine (SVM) to combine the selected factors to construct a clinical model (Model^C^), a 2D model (Model^2D^), and a 3D model (Model^3D^). Since the SVM could not directly predict the risk of overt HE, a probability calibration with isotonic regression was used to predict the risk. Third, we compared the discrimination and calibration of Model^2D^ and Model^3D^ to prove the advantages of 3D segmentation. Fourth, the selected clinical and 3D factors were combined to construct Model^C-3D^. We then compared the discrimination and calibration of Model^C^, Model^3D^, and Model^C-3D^. During the abovementioned steps, discrimination was tested by the Delong test, net reclassification improvement (NRI), and integrated discrimination improvement (IDI), with calibration compared using calibration plots. Finally, for the best model, we performed decision curve analysis (DCA) to show the net benefit, with an applet constructed to facilitate future application. We also performed subgroup analysis to test its performance in different patient subgroups (Fig. 2c).

All algorithms involving features and model building were implemented using Python (version 3.6), and all statistical analyses were performed in R (version 4.2.1). SVM was performed by using the “scikit-learn” package (https://scikit-learn.org/stable/). All statistical tests were two-sided, and *p* < 0.050 was considered statistically significant. The report of our study strictly followed the Transparent Reporting of a multivariable prediction model for Individual Prognosis or Diagnosis statement.

## Results

### Study population and baseline

A total of 487 patients were included in our study. They were divided into a training dataset (390 patients from three hospitals) and an external validation dataset (97 patients from another two hospitals). Symptoms leading to TIPS included variceal bleeding (390 patients; training: 336 patients; validation: 54 patients) and refractory ascites (97 patients; training: 77 patients; validation: 20 patients). There were no statistical differences between the training and validation datasets regarding the demographic factors. The baseline characteristics of the patients are reported in Table 1. Overt HE occurred in 152 patients (training: 101 patients; validation: 51 patients) (Supplementary Table 5).

**Table 1:** Baseline demographics of patients

Clinical factors	Training dataset (N = 390)	Validation dataset (N = 97)	*p* Value
Age (year)	51.8 ± 11.8	55.2 ± 14.5	0.012*
Sex (N)			0.062
Male	308	68	
Female	82	29	
Etiology (N)			0.820
Alcohol	163	26	
Hepatitis B/C	144	47	
Cholestatic	11	1	
Others	72	23	
Child–Pugh score (point)	8 (6, 9)	7 (7, 9)	0.880
ALT	20.0 (14.0, 30.0)	21.0 (15.0, 36.0)	0.222
AST	28.0 (22.0, 41.0)	29.0 (21.5, 46.5)	0.177
Direct bilirubin (μmol/L)	8.7 (5.5, 14. 2)	9.6 (6.0, 17.7)	0.122
Indirect bilirubin (μmol/L)	9.1 (6.4, 13.4)	10.0 (7.1, 16.8)	0.216
Serum sodium (mmol/L)	140.0(138.0, 142.0)	139.0 (136.0, 142.0)	0.015*
INR	1.3 (1. 2, 1.5)	1.3 (1.2, 1.4)	0.497
Ammonia (μmol/L)			0.552
< 72.0	349	85	
≥ 72.0	41	12	
Indication for TIPS (N)			0.096
Variceal bleeding	336	77	
Refractory ascites	54	20	
Liver cancer			0.961
Yes	53	13	
No	337	84	
Diabetes			0.764
Yes	83	22	
No	307	75	

Normally distributed factors are expressed using means ± standard deviations; non-normally distributed factors are expressed as medians (interquartile ranges)

*ALT* Alanine aminotransferase, *AST* Aspartate aminotransferase, *INR* International normalized ratio

*With a *p* < 0.050

### Model construction

After LASSO regression, 1394 preliminary factors were identified, including 20 clinical factors, three vascular factors, 488 2D factors (nine morphologic factors and 479 high-dimensional factors), and 883 3D factors (20 morphologic factors and 863 high-dimensional factors). After SVM, five clinical factors, two vascular factors, six 2D factors, and seven 3D factors were used to construct Model^C^, Model^2D^, Model^3D^, and Model^C-3D^ (Supplementary Table 6 and Supplementary Table 7).

### Comparison between the Model^2D^ and Model^3D^

When the 2D and 3D models were compared, Model^3D^ had a better area under the curve (AUC) than did Model^2D^ in the training (AUC = 0.719 vs. 0.691, Supplementary Fig. 1-a) and validation (AUC = 0.730 vs. 0.622, Supplementary Fig. 1-b) datasets, with the result of Delong test, NRI, and/or IDI displayed in Supplementary Table 8. For calibration, Model^3D^ and Model^2D^ displayed similar curves (Supplementary Fig. 1-c, 1-d). Based on these results, we used 3D factors to construct the combined model.

### Comparison among the Model^C^, Model^3D^, and Model^C-3D^

**Fig. 3:**
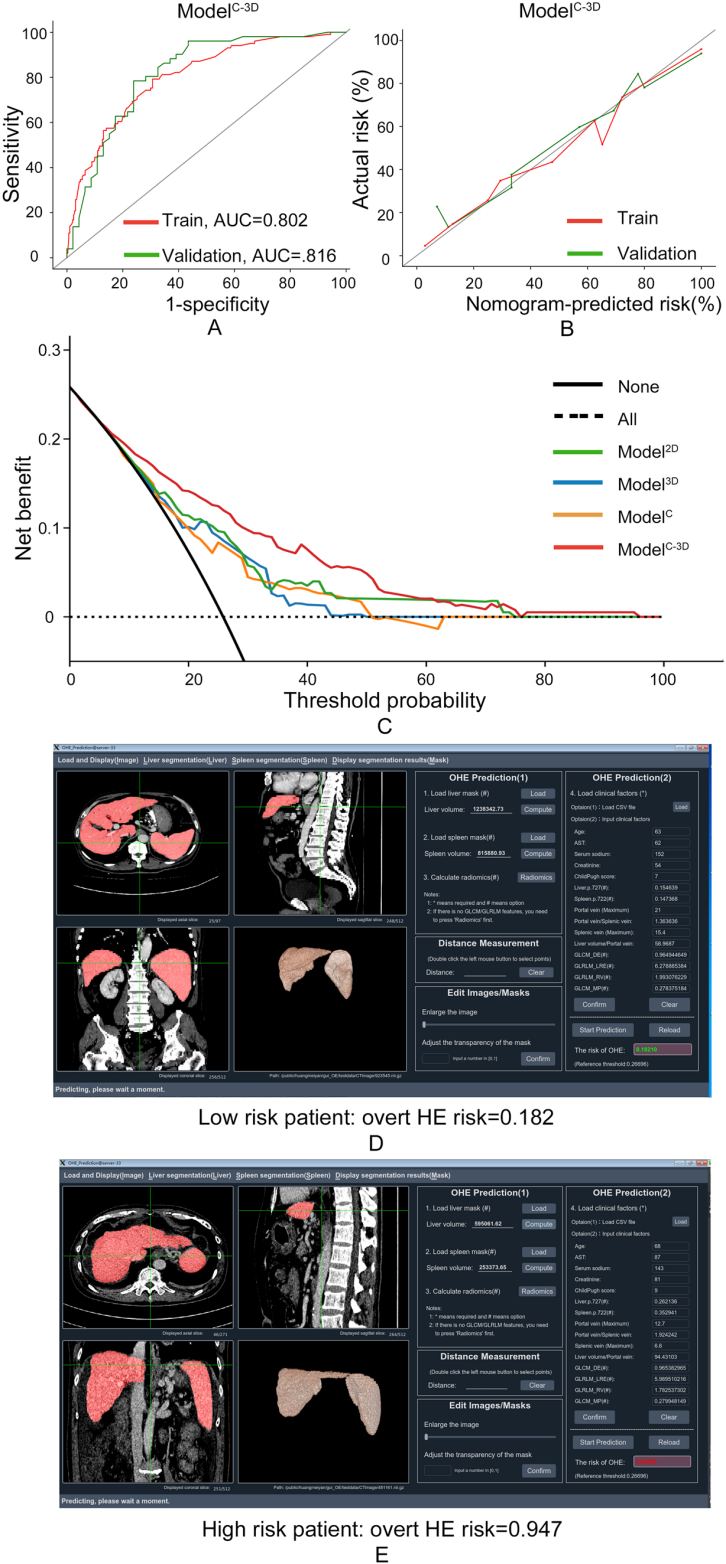
Performance of the combined model and its applet. Performance evaluation of the combined model and its applet. **a** Receiver operating characteristic (ROC) curve analysis showing the area under the curve (AUC) for the combined model in the training dataset (AUC: 0.802) and validation dataset (AUC: 0.816). **b** Calibration plot demonstrating the performance of the combined model. **c** Decision curve analysis (DCA) comparing the combined model with three other models. Patient examples classified as **d** low risk and **e** high risk by the combined model

When the clinical, 3D, and combined models were compared, Model^C-3D^ had a better AUC than Model^C^ and Model^3D^ in both datasets (training: 0.802 vs. 0.735 vs. 0.719; validation: 0.816 vs. 0.723 vs. 0.730; Supplementary Fig. 2-a, 2-b), with statistical differences regarding the Delong test, NRI, and/or IDI (Supplementary Table 8). Regarding calibration, Model^C-3D^ performed better than Model^C^ and Model^3D^ (Supplementary Fig. 2-c, 2-d). Based on these results, Model^C-3D^ was identified as the optimal model, with its confusion matrix and DCA curve displayed (Supplementary Fig. 3, Fig. 3c).

### Subgroup analysis

**Fig. 4:**
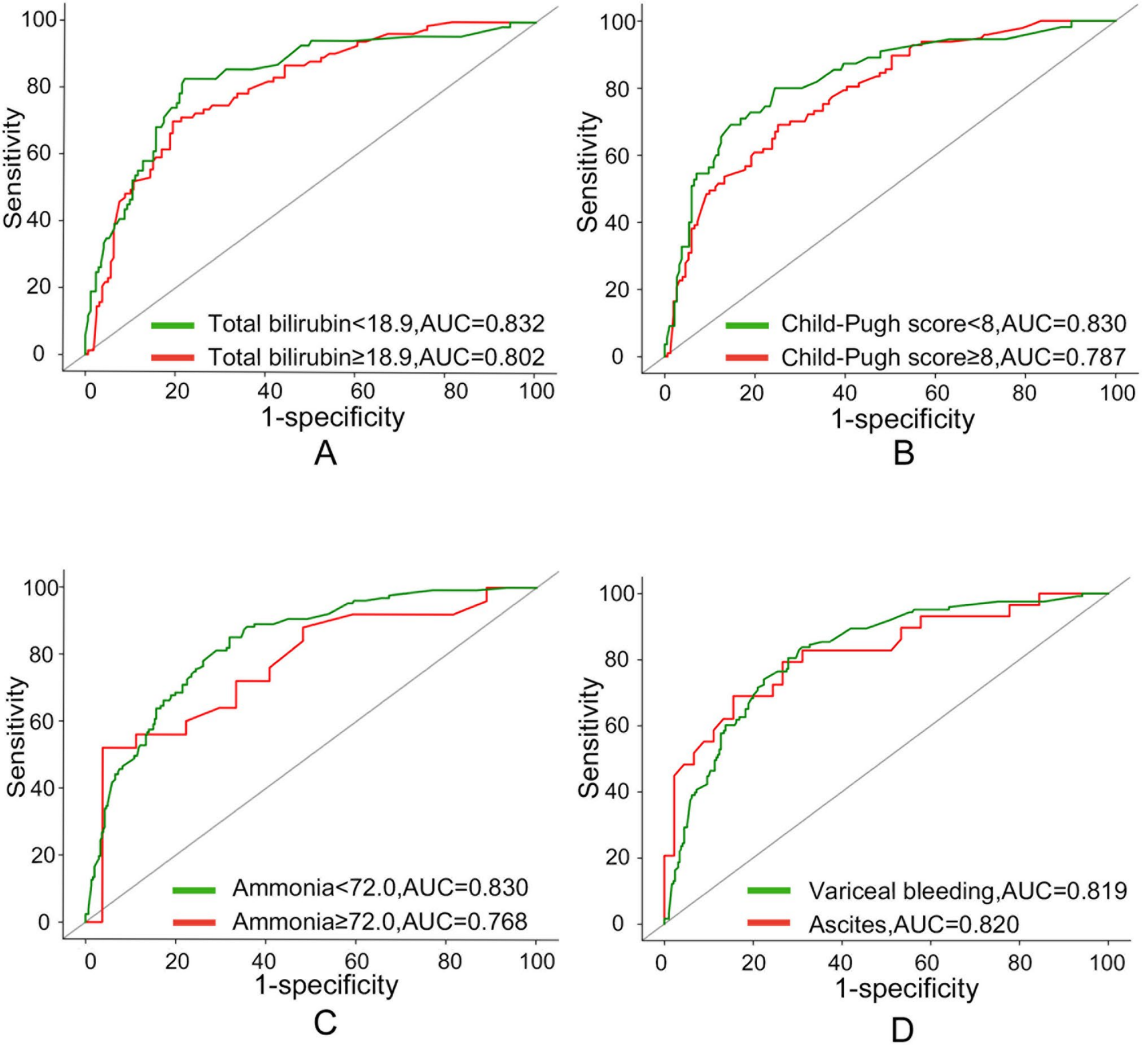
Subgroup analysis of Model^C-3D^. The performance of the Model^C-3D^ was not influenced by **a** the total bilirubin level, **b** Child–Pugh score, **c** ammonia level, and **d** the indication for TIPS. TIPS: transjugular intrahepatic portosystemic shunt

To assess comparability among different subgroups, we tested whether total bilirubin (TBIL) level, Child–Pugh score, ammonia level, and the indication for TIPS could influence the performance of Model^C-3D^. The results showed that no statistical difference existed among the subgroups (Supplementary Table 9): total bilirubin < 18.9 vs. total bilirubin ≥ 18.9 (0.832 vs. 0.802, *p* = 0.478; Fig. 4a); Child–Pugh score < 8 vs. Child–Pugh score ≥ 8 (0.830 vs. 0.787; *p* = 0.322; Fig. 4b); preoperative ammonia < 72.0 vs. preoperative ammonia ≥ 72.0 (0.830 vs. 0.787; *p* = 0.383; Fig. 4c); and variceal bleeding vs. refractory ascites (0.819 vs. 0.820; *p* = 0.995; Fig. 4d). Accordingly, an applet for Model^C-3D^ was constructed, and patients identified as low-risk (Fig. 3d) and high-risk (Fig. 3e) were displayed. (https://drive.google.com/drive/folders/15WQae0MyRt61ND0KUI8UzcnzZ5ORX0e_).

## Discussion

**Fig. 5:**
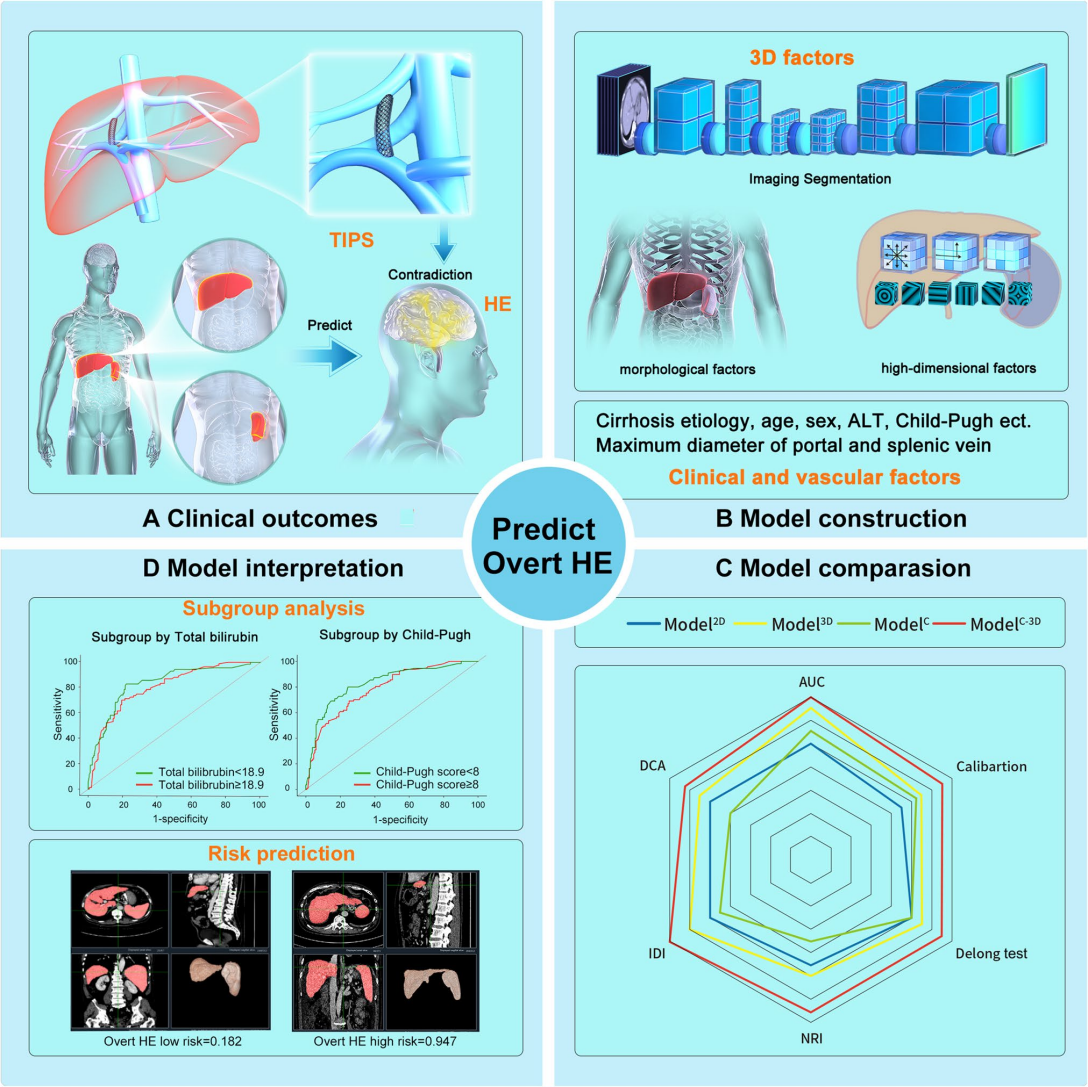
Study design. **a** The objective of this study was to predict the occurrence of post-transjugular intrahepatic portosystemic shunt (TIPS) overt hepatic encephalopathy (HE). **b** Data were collected on various factors, including clinical, vasculature, 2D, and 3D factors (including morphologic and high-dimensional factors) as candidate variables. **c** Model comparisons were performed, initially comparing 2D and 3D models, followed by a comparison between a clinical model (Model^C^), 3D model (Model^3D^), and a combined model (Model^C-3D^) to identify the optimal model. **d** Subgroup analysis was conducted for the optimal model, and an applet was developed for future clinical application. *AUC* area under the curve, *NRI* net reclassification improvement, *IDI* integrated discrimination improvement, *TIPS* transjugular intrahepatic portosystemic shunt

For the prediction of post-TIPS overt HE, 3D-based assessment for the liver and the spleen significantly improved the performance of the model for both discrimination and calibration (Fig. 5). Based on these results, the 3D-based assessment could be used to assist patient selection for TIPS; furthermore, it might provide additional information for studies related to TIPS, and even cirrhosis.

First-line treatments for variceal bleeding and ascites caused by portal hypertension include large-volume paracentesis [27] and non-selective β-blockers [28]; TIPS is an alternative option [1]. Since the pressure in the portal vein is not controlled, patients who accept first-line treatment have a high recurrence rate for variceal bleeding and ascites. However, considering the risk of post-TIPS overt HE, the guidelines recommend TIPS only as a second-line treatment [4–6]. Previously, we demonstrated that post-TIPS overt HE could be predicted [22, 23], which could allow more patients to accept TIPS. However, the results could only be internally validated with a small sample size. More importantly, the 2D-based manual measurement had several limitations: potential bias due to slice selection, concerns about comprehensiveness and representation, time and labor costs, and the lack of assessment of cirrhosis-impacted organs other than the liver (such as the spleen). Considering the need to predict post-TIPS overt HE, we conducted this study with a 3D assessment of both the liver and the spleen and with external validation, aiming to construct a more elaborate and precise model.

Automatic segmentation and data extraction were key steps to perform 3D assessments of the liver and the spleen. For automatic segmentation, deep learning is a promising pathway. However, the performance of deep learning methods largely depends on the preprocessing methods, model structure, and training strategy [29]. Compared to other frameworks, nnU-Net can automatically achieve the optimal configuration of the aforementioned settings to obtain excellent results [22], and has been widely used in similar studies [19]. For data extraction, parameters based on the traditional visual system can provide information on morphological changes. Meanwhile, high-dimensional data, such as radiomic features, can provide information overlooked by traditional methods [30]. Therefore, we combined these two sets of data in this study.

As expected, 3D segmentation achieved sufficient precision for the assessment. The 3D model (Model^3D^) outperformed Model^2D^ in discrimination, especially in the validation dataset (0.730 vs. 0.622). However, it did not achieve sufficient calibration. Meanwhile, the clinical model had unsatisfactory discrimination (0.735 in the training dataset and 0.723 in the validation dataset). Thus, we combined the clinical and 3D factors. The combined Model^C-3D^ demonstrated significantly improved discrimination and calibration. These results showed that the technological improvement from 2D assessment to 3D assessment was essential and that a combination of clinical and radiological factors remained a reasonable means to construct a precise model to predict post-TIPS overt HE.

In the combined model (Model^C-3D^), the clinical factors included age [1], serum sodium [23], aspartate aminotransferase [5], creatinine [5], and the Child–Pugh score [1], consistent with the previous study. Parameters related to morphological changes in the liver and the spleen vasculature, such as the maximum diameter of the portal vein and the ratio of the maximum diameters of the portal and splenic veins, remained significant [22].

For morphological 3D factors, the identified parameters could be classified into two categories. The first category included parameters reflecting volume-related changes, including the liver volume standardized by the maximum diameter of the portal vein. As expected, liver volume alone was not significant in predicting overt HE, possibly due to physiological differences, such as somatotype-related liver enlargement. However, if the liver and its related vasculature did not enlarge synchronously, it was more likely because of pathological rather than physiological reasons. Thus, standardization by organ-specific vasculature (such as liver volume by the maximum diameter of the portal vein or spleen volume by the maximum diameter of the splenic vein) was reasonable and necessary to assess volume. The second category included parameters correlated with changes in CT attenuation. Considering that CT attenuation could be highly subject to the time of image capture, median or mean CT attenuation might fail to reflect the pathological change. The change rate of CT attenuation could be used to control this bias. However, when accidental extremum existed, the variation range could be influenced; therefore, the change rate measured by the interquartile range was a more reasonable method. As expected, the maximum gradient CT attenuation of the liver and the minimum gradient CT attenuation of the spleen were significant in predicting overt HE, which proved our hypothesis to be reasonable.

For high-dimensional 3D factors, the identified radiomic features could also be classified into two categories: grey-level run length matrix (GLRLM)) features for local heterogeneity and grey-level co-occurrence matrix (GLCM) features for regional heterogeneity [31]. The GLRLM features included RunVariance and LongRunEmphasis, while the GLCM features included MaximumProbability and DifferenceEntropy. The GLRLM heterogeneity analysis was grouped in a single matrix. Therefore, in our GLRLM, each cell GLRLM (*i, j*) corresponded to the run number of length *j* formed by voxels of intensity in box *i* in all directions. The GLCM was a square matrix of the size of the gray levels present in the image; it was defined by the distance between voxels, e.g., adjacent voxels or voxels having at least one common neighbor. Finally, a set of two GLRLM and two GLCM heterogeneous textural features was used in our study.

We have not included the presence of major portosystemic shunts as factors in our model. In our previous research [22, 23], we found that major portosystemic shunts had limited effect on hepatic encephalopathy. Compared with our previous studies [22, 23], this study improved 3D segmentation and data extraction. However, there were still some limitations. First, considering the differences between Eastern and Western patients, e.g., viral cirrhosis and alcoholic cirrhosis, validation in a Western cohort may be necessary before our model can be applied to Western patients. Second, limited by the retrospective design, our study could not assess occult HE, which should also be studied in future prospective studies. Third, although the liver and the spleen were automatically segmented in 3D slices, their related vasculature was manually segmented in 2D slices. This was because we had not solved the imbalance problems: foreground (vascular system) vs. background (other regions such as liver parenchyma, stomach, etc.), and thin vs. thick vessels. With continued efforts, we might be able to perform accurate automatic 3D segmentation for the vascular system in the future. Fourth, patients without an 8-mm polytetrafluoroethylene-covered stent were excluded to control the possible confounding factors. Therefore, whether our conclusion is applicable to patients with a 10-mm polytetrafluoroethylene-covered stent or bare metal stent requires further exploration.

In conclusion, we constructed a model that could predict post-TIPS overt HE based on a 3D assessment of the liver and the spleen. Assisted by our model, patients with low risk may be able to accept alternative TIPS treatment. Moreover, the 3D factors (including morphological and high-dimensional factors) demonstrated multiple advantages, which can be used for future studies related to TIPS.

### Data sharing

Owing to privacy concerns, the data related to patients are not available for public access but can be obtained from the corresponding author on reasonable request, subject to approval by the institutional review board of Zhuhai People’s Hospital.

### Supplementary Information

Below is the link to the electronic supplementary material.

Supplementary file1 (DOCX 10020 KB)

## Data Availability

It can be obtained from the corresponding author on reasonable request.
