# Supplementary material for: 3D automatic liver and spleen assessment in predicting overt hepatic encephalopathy before TIPS: a multi-center study
Source: Hepatol Int. 2023 Aug 2;17(6):1545–56. doi: 10.1007/s12072-023-10570-5 (PMC10661776; doi:10.1007/s12072-023-10570-5)
Supplement: Supplementary file 1 — Supplementary file1 (DOCX 10020 KB) [file 12072_2023_10570_MOESM1_ESM.docx]

**3D automatic liver and spleen assessment in predicting overt hepatic encephalopathy before TIPS: A multi-center study**

Xiaoqiong Chen, MD *; Tao Wang, PhD *; Zhonghua Ji, MD *; Junyang Luo, MD *; Weifu Lv, MD; Haifang Wang, MD; Yujie Zhao, MD; Chongyang Duan, PhD; Xiangrong Yu, MD; Qiyang Li, MD; Jiawei Zhang, PhD; Jinqiang Chen, MD; Xiaoling Zhang, PhD; Mingsheng Huang, MD; Shuoling Zhou, PhD; Ligong Lu, MD ^#^; Meiyan Huang, PhD ^#^; Sirui Fu, MD ^#^.

**Supplementary material**

**Table of contents**

1. Supplementary Table 1. Clinical characterization of overt HE
2. Supplementary Table 2. Major parameters of CT image acquisition
3. Supplementary Text 1: 3D segmentation methodology
4. Supplementary Table 3. Candidate clinical and vascular factors
5. Supplementary Table 4. Candidate morphological factors
6. Supplementary Table 5. Baseline demographics of patients (about overt HE)
7. Supplementary Table 6. Identified factors for model construction
8. Supplementary Table 7. Coefficient of identified factors for Model^C-3D^
9. Supplementary Fig. 1. Comparison between the Model^2D^ and Model^3D^
10. Supplementary Fig. 2. Comparison among the Model^C^, Model^3D^ and Model^C-3D^
11. Supplementary Fig. 3. The AUC of Model^C-3D^ by the confusion matrix
12. Supplementary Table 8. Pairwise comparison of the models
13. Supplementary Table 9. Subgroup analysis of the AUCs
14. **Supplementary Table 1. Clinical characterization of overt HE**

Supplementary Table 1. Clinical characterization of overt HE

| Grade | Clinical characterization |
| --- | --- |
| II | increased fatigue, apathy, flapping tremor/asterixis, ataxia, slurred speech |
| III | somnolence, marked disorientation, rigor, stupor |
| IV | coma |

1. **Supplementary Table** **2**

| **Supplementary Table 2.** **Major parameters of CT image acquisition** | | | | | | | | | |
| --- | --- | --- | --- | --- | --- | --- | --- | --- | --- |
| **Hospitals** | **Scanner** | **TV (kV)** | **TC (mA)** | **RT (s)** | **DC (mm)** | **FOV (mm)** | **PM** | **Reconstruction** | **ST (mm)** |
| **NFH** | Philips Brilliance | 120 | 142 | 0.75 | 128×0.625 | 300×300 | 512×512 | Filter sharp (C) | 5 mm |
| **SPH** | Philips Brilliance | 120 | 250 | 0.50 | 64×0.625 | 500×500 | 1024×1024 | Filter sharp (C) | 5 mm |
| **SYSUTAH** | Toshiba Aquilion | 120 | 250 | 0.50 | 320x0.500 | 500×500 | 512×512 | Filter Standard(A) | 1 mm |
| **STCUAPH** | GE Optima | 120 | 280 | 0.80 | 64×0.625 | 400×400 | 512×512 | Filter Standard(B) | 5 mm |
| **ZPH** | Siemens Somatom  Definition Flash | 120 | 160 | 0.50 | 64×0.625 | 350×350 | 512×512 | Filter sharp (C) | 2 mm  5 mm |
| TV: tube voltage; TC: tube current; RT: rotation time; DC: detector collimation; FOV: field of view; PM: pixel matrix; ST: slice thickness; NFH: Nanfang Hospital; SPH: Shenzhen People’s Hospital; SYSUTAH: The Third Affiliated Hospital of Sun Yat-sen University; STCUAPH: The First Affiliated Hospital of the University of Science and Technology of China; ZPH: Zhuhai People’s Hospital | | | | | | | | | |

1. **Supplementary Text 1: 3D segmentation methodology**

All the image data was exported in DICOM format for image 3D segmentation and data extraction. nnU-Net is a widely used segmentation framework in deep learning. Thus, two nnU-Nets, with the same architecture, were utilized to segment the liver and the spleen. However, since the training of deep learning relies on large samples, a special learning strategy was applied. Initially, 100 segmented CT scans delineated by radiologists were used to train the nnU-Nets. Next, a batch of rest CT scans were fed to the nnU-Nets to obtain the segmentation results, and they were checked and fine-tuned by radiologists. Then, the fine-tuned data were used as new samples to retrain the nnU-Nets to improve performance. This process was repeated several times; the nnU-Nets were forced to evolve via our learning strategy until they were satisfactory. Finally, the trained nnU-Nets were applied to achieve the automatic segmentation of rest CT scans.

1. **Supplementary Table 3**

| Supplementary Table 3. Candidate clinical and vascular factors | |
| --- | --- |
| Classification | **Factor name** |
| Clinical factors |  |
|  | Age |
|  | Sex |
|  | Indication for TIPS |
|  | Diabetes |
|  | Cirrhosis etiology |
|  | Accompanying liver cancer |
|  | Hemoglobin |
|  | Platelet count |
|  | Aspartate aminotransferase |
|  | Alanine aminotransferase |
|  | Direct bilirubin |
|  | Indirect bilirubin |
|  | Serum sodium |
|  | Creatinine |
|  | Urea nitrogen |
|  | International normalized ratio |
|  | Activated partial thromboplastin time |
|  | Thrombin time |
|  | Ammonia |
|  | Child-Pugh score |
| Vascular factors |  |
|  | Maximum diameter of the portal vein |
|  | Maximum diameter of the splenic vein |
|  | Diameter ratio of the portal and splenic veins |

1. **Supplementary Table 4**

| **Supplementary Table 4.** **Candidate morphological factors** | | |
| --- | --- | --- |
| **Classification of changes** | **2D factor** | **3D factor** |
| **Diameter or volume** |  |  |
|  | Maximum diameter of the liver | Liver volume |
|  | Ratio between the maximum diameters of the liver and the portal vein | Ratio between the liver volume and the maximum diameter of the portal vein |
|  | Positive liver depression number^1^ | Liver depression volume^2^ |
|  |  | Ratio between liver depression and liver volume |
|  | Maximum diameter of the spleen | Spleen volume |
|  | Ratio between the maximum diameters of the spleen and the splenic vein | Ratio between spleen volume and the maximum diameter of the splenic vein |
|  | Ratio between the maximum diameters of the liver and the spleen | Ratio between the liver and spleen volumes |
| **CT** **attenuation** |  |  |
|  | CT attenuation of the liver | Mean CT attenuation of the liver |
|  |  | Median CT attenuation of the liver |
|  |  | Interquartile CT attenuation of the liver |
|  |  | Minimum gradient CT attenuation of the liver^2^ |
|  |  | Maximum gradient CT attenuation of the liver^3^ |
|  | CT attenuation of the spleen | Mean CT attenuation of the spleen |
|  |  | Median CT attenuation of the spleen |
|  |  | Interquartile CT attenuation of the spleen |
|  |  | Minimum gradient CT attenuation of the spleen^2^ |
|  |  | Maximum gradient CT attenuation of the spleen^3^ |
|  | CT attenuation ratio between the liver and the spleen | Mean CT attenuation ratio between the liver and the spleen |
|  |  | Median CT attenuation ratio between the liver and the spleen |
|  |  | Interquartile CT attenuation ratio between the liver and the spleen |
| ^1^Positive liver depression was defined as depression ≥ 5 mm; ^2^Calculated by (P75-P25)/P25; ^3^Calculated by (P75-P25)/P75  ^2^To obtain the volume of liver nodularity, we adopted the following strategies: (a) smoothing the liver surface first; (b) directly calculating the volume difference between the smoothed liver and the original liver. Smoothing process: after extracting the liver surface curve, sparse sampling was performed on the points of the curve, and spline interpolation was performed on the sparsely sampled points to obtain a smooth surface. | | |

1. **Supplementary Table 5**

| **Supplementary Table 5. Baseline demographics of patients (about overt HE)** | | | |
| --- | --- | --- | --- |
| **Clinical**  **factors** | **Without overt HE**  **(N=335)** | **With overt HE**  **(N=152)** | ***P*-**  **value** |
| **Age** (year) | 51.2 ± 12.0 | 55.3 ± 11.1 | <0.001* |
| **Sex** (N) |  |  | 0.701 |
| Male | 257 | 118 |  |
| Female | 78 | 34 |  |
| **Etiology** (N) |  |  | 0.307 |
| Alcohol | 124 | 67 |  |
| Hepatitis B/C | 136 | 53 |  |
| Cholestatic | 7 | 5 |  |
| Others | 68 | 27 |  |
| **Child-Pugh score** (point) | 7 (6, 8) | 8 (7, 9) | <0.001* |
| **ALT** (U/L) | 20.0 (14.0, 29.0) | 22.0 (15.0, 37.0) | 0.020* |
| **AST** (U/L) | 28.0 (21.5, 40.0) | 31.5 (23.0, 51.5) | 0.010* |
| **Direct bilirubin (**μmol/L**)** | 8.6 (5.4, 14. 0) | 9.8 (6.0, 16.3) | 0.059 |
| **Indirect bilirubin (**μmol/L**)** | 9.2 (6.6, 13.6) | 9.7 (6.6, 13.7) | 0.913 |
| **Serum sodium** (mmol/L) | 140.0(138.0, 142.0) | 139.0 (136.0, 141.0) | <0.001* |
| **INR** | 1.3 (1.2, 1.4) | 1.3 (1.2, 1.5) | 0.040* |
| **Ammonia** (μmol/L) |  |  | 0.014* |
| < 72.0 | 307 | 128 |  |
| ≥ 72.0 | 28 | 24 |  |
| **Indication for TIPS** (N) |  |  | 0.429 |
| Variceal bleeding | 287 | 126 |  |
| Refractory ascites | 48 | 26 |  |
| **Liver cancer** (N) |  |  | 0.123 |
| Yes | 40 | 26 |  |
| No | 295 | 126 |  |
| **Diabetes** (N) |  |  | 0.050 |
| Yes | 64 | 41 |  |
| No | 271 | 111 |  |
| * With a *P* < 0.050  Normally distributed factors are expressed using means ± standard deviations; non-normally distributed factors are expressed as medians (interquartile ranges).  ALT: Alanine aminotransferase; AST: Aspartate aminotransferase; INR: International normalized ratio. | | | |

1. **Supplementary Table 6**

**Supplementary Table 6. Identified factors for model construction**

| **Classification** | **Factor** |
| --- | --- |
| **Clinical factors** |  |
|  | Age |
|  | Serum sodium |
|  | Aspartate aminotransferase |
|  | Creatinine |
|  | Child-Pugh score |
| **Vascular factors** |  |
|  | Maximum diameter of the portal vein |
|  | Maximum diameter ratio of the portal and the splenic veins |
| **2D factors** |  |
| **Morphological** |  |
|  | Ratio between the maximum diameters of the liver and the portal vein |
|  | Ratio between the maximum diameters of the spleen and the splenic vein |
|  | Ratio between CT attenuation of the liver and the spleen |
| **High-dimensional** |  |
|  | Original_glrlm_RunLengthNonUniformityNormalized |
|  | Wavelet-HL_glcm_Imc2 |
|  | Wavelet-HL_glcm_SumEntropy |
| **3D factors** |  |
| **Morphological** |  |
|  | Ratio between the liver volume and the maximum diameter of the portal vein |
|  | Maximum gradient CT attenuation of the liver^1^ |
|  | Minimum gradient CT attenuation of the spleen ^2^ |
| **High-dimensional** |  |
|  | Wavelet-LHH_glrlm_RunVariance |
|  | Wavelet-HLH_glcm_MaximumProbability |
|  | Wavelet-LHH_glrlm_LongRunEmphasis |
|  | Wavelet-LHL_glcm_DifferenceEntropy |
| ^1^Calculated by (P75-P25)/P75; ^2^Calculated by (P75-P25)/P25. | |

1. **Supplementary Table 7. Coefficient of identified factors for Model^C-3D^**

**Supplementary Table 7. Coefficient of identified factors for Model^C-3D^**

| **Classification** | **Factor** | **Coefficient** |
| --- | --- | --- |
| **Clinical factors** |  |  |
|  | Age | 0.364 |
|  | Serum sodium | 0.177 |
|  | Aspartate aminotransferase | 0.071 |
|  | Creatinine | 0.225 |
|  | Child-Pugh score | 0.231 |
| **Vascular factors** |  |  |
|  | Maximum diameter of the portal vein | 0.242 |
|  | Maximum diameter ratio of the portal and the splenic veins | 0.565 |
| **3D factors** |  |  |
| **Morphological** |  |  |
|  | Ratio between the liver volume and the maximum diameter of the portal vein | 0.221 |
|  | Maximum gradient CT attenuation of the liver^1^ | 0.272 |
|  | Minimum gradient CT attenuation of the spleen^2^ | 0.043 |
| **High-dimensional** |  |  |
|  | Wavelet-LHH_glrlm_RunVariance | 0.024 |
|  | Wavelet-HLH_glcm_MaximumProbability | 0.190 |
|  | Wavelet-LHH_glrlm_LongRunEmphasis | 0.358 |
|  | Wavelet-LHL_glcm_DifferenceEntropy | 0.398 |

^1^Calculated by (P75-P25)/P75; ^2^Calculated by (P75-P25)/P25

1. **Supplementary Fig. 1. Comparison between the Model^2D^ and Model^3D^**

**
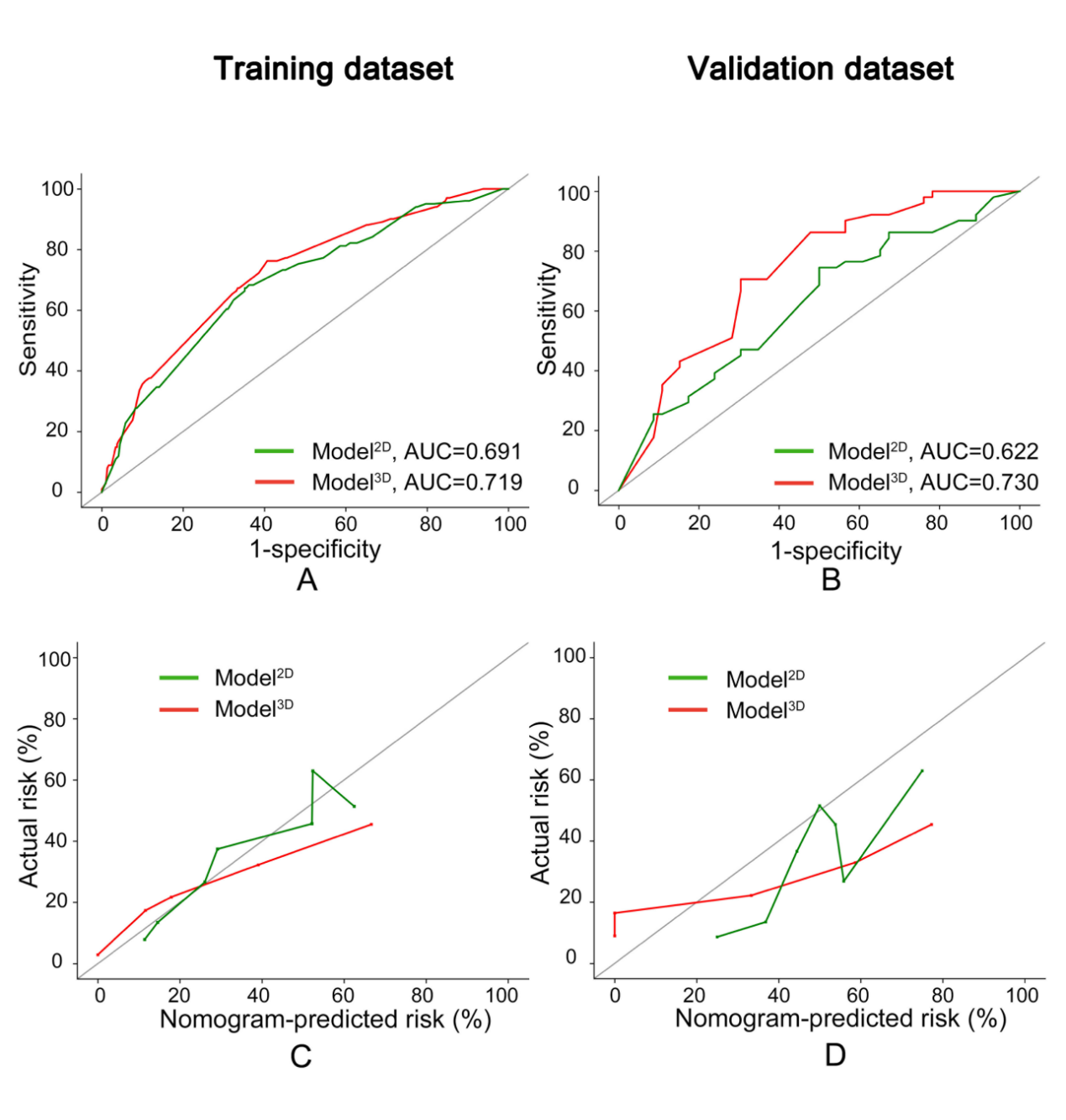
**

**Supplementary Fig. 1. Comparison between the Model^2D^ and Model^3D^**

To predict post-TIPS overt HE, the AUCs of the 2D and 3D models for (A) the training dataset and (B) the validation dataset are shown. The calibration of (C) the training dataset and (D) the validation dataset are shown.

1. **Supplementary Fig. 2. Comparison among the Model^C^, Model^3D^ and Model^C-3D^**


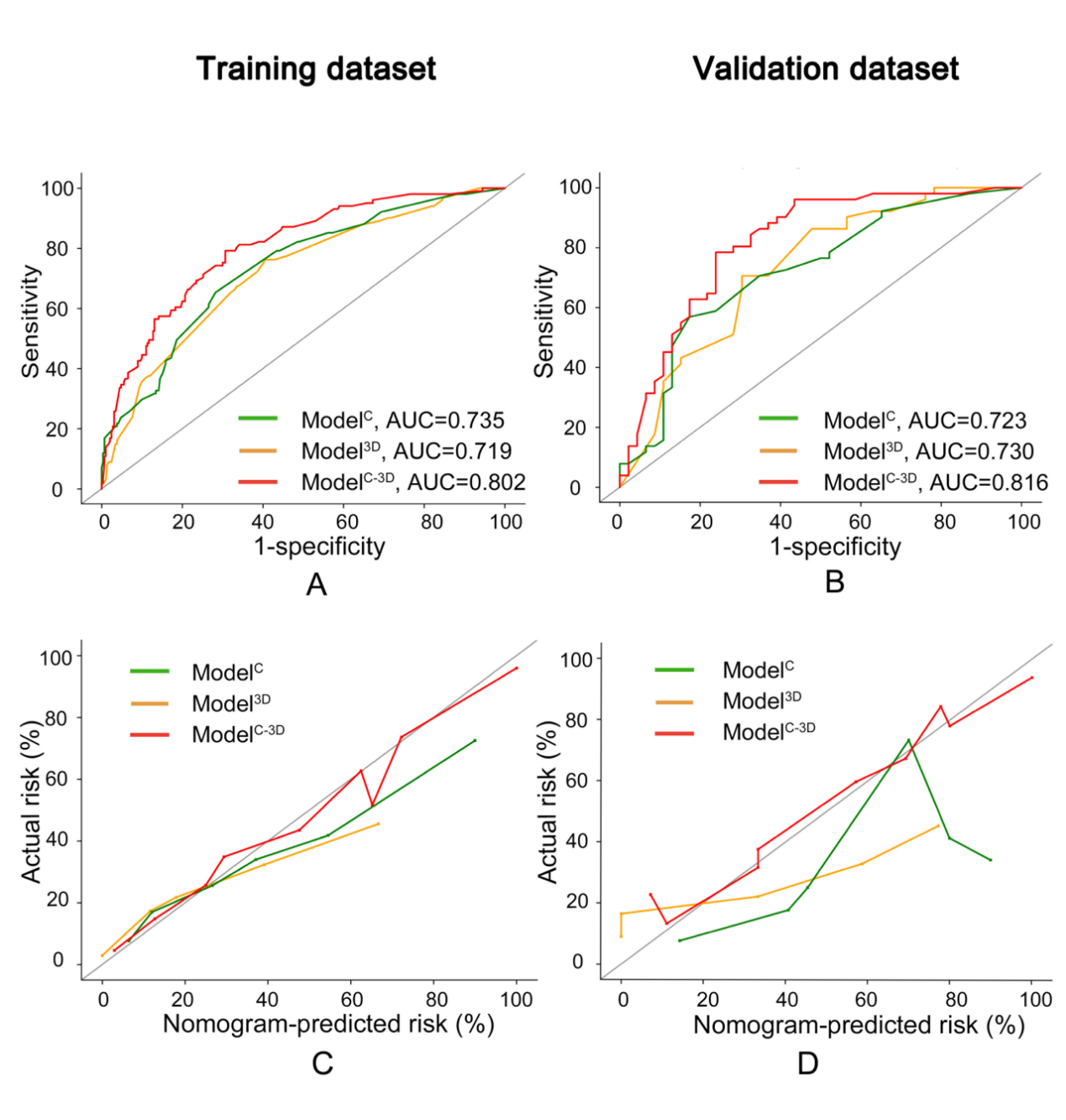


**Supplementary Fig. 2. Comparison among the Model^C^, Model^3D^ and Model^C-3D^**

To predict post-TIPS overt HE, the AUCs of the 3D, clinical, and combined models are shown for (A) the training dataset and (B) the validation dataset. The calibration of (C) the training dataset and (D) the validation dataset are shown.

1. **Supplementary Fig. 3. The AUC of Model^C-3D^ by the confusion matrix**


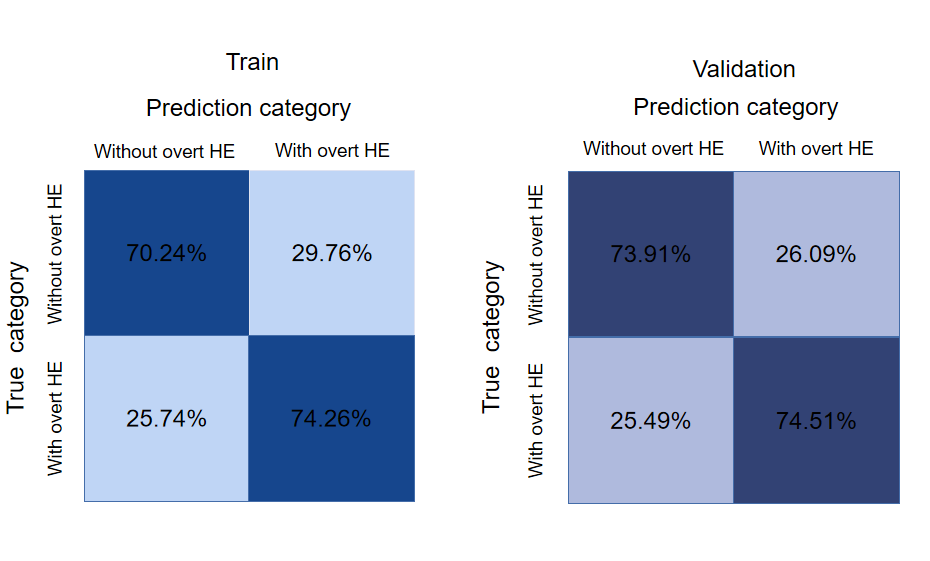


**Supplementary Fig. 3. The AUC of Model^C-3D^ by the confusion matrix.**

The confusion matrix of the training and validation datasets. (A) In the train, the sensitivity of predicting the post-TIPS overt HE was 74.26% and specificity was 70.24%. (B) in the validation, the sensitivity of predicting the post-TIPS overt HE was 74.51% and specificity was 73.91%.

1. **Supplementary Table 8**

| **Supplementary Table** **8. Pairwise comparison of the models** | | | |
| --- | --- | --- | --- |
|  | **Delong test** | **NRI^1^** | **IDI^2^** |
| **Training dataset** |  |  |  |
| Model^2D^ vs. Model^3D^ | 0.473 | 0.015* | 0.050 |
| Model^C^ vs. Model^3D^ | 0.668 | 0.028* | 0.017* |
| Model^3D^ vs. Model^C-3D^ | 0.001* | <0.001* | <0.001* |
| Model^C^ vs. Model^C-3D^ | <0.001* | <0.001* | <0.001* |
| **Validation dataset** |  |  |  |
| Model^2D^ vs. Model^3D^ | 0.089 | 0.467 | 0.942 |
| Model^C^ vs. Model^3D^ | 0.916 | 0.688 | 0.461 |
| Model^3D^ vs. Model^C-3D^ | 0.019* | 0.002* | <0.001* |
| Model^C^ vs. Model^C-3D^ | 0.010* | <0.001* | <0.001* |
| Data are presented as *P* values. * (*P* < 0.050 is significant)  ^1^NRI: net reclassification improvement; ^2^IDI: integrated discrimination improvement. | | | |

1. **Supplementary Table 9**

| **Supplementary Table 9. Subgroup analysis of the AUCs** | | |
| --- | --- | --- |
| **Subgroups divided by** | **Z** | ***P* value** |
| **Total bilirubin** (< 18.9 μmol/L vs. ≥ 18.9 μmol/L) | 0.710 | 0.478 |
| **Child-Pugh score (**< 8 vs. ≥ 8**)** | 0.992 | 0.322 |
| **Ammonia** (< 72.0 μmol/L vs. ≥ 72.0 μmol/L) | 0.879 | 0.383 |
| **Indication for TIPS (**variceal bleeding vs. ascites**)** | 0.006 | 0.995 |
